# Interactions between *Verticillium dahliae* and cotton: pathogenic mechanism and cotton resistance mechanism to Verticillium wilt

**DOI:** 10.3389/fpls.2023.1174281

**Published:** 2023-04-21

**Authors:** Yutao Zhu, Mei Zhao, Taotao Li, Lianzhe Wang, Chunli Liao, Dongxiao Liu, Huamin Zhang, Yanpeng Zhao, Lisen Liu, Xiaoyang Ge, Bingbing Li

**Affiliations:** ^1^ College of Life Science and Engineering, Henan University of Urban Construction, Pingdingshan, China; ^2^ Zhengzhou Research Base, State Key Laboratory of Cotton Biology, School of Agricultural Sciences, Zhengzhou University, Zhengzhou, China; ^3^ State Key Laboratory of Cotton Biology, Institute of Cotton Research, Chinese Academy of Agricultural Sciences, Anyang, China

**Keywords:** cotton, Verticillium wilt, *Verticillium dahliae*, pathogenic mechanism, resistance mechanism

## Abstract

Cotton is widely grown in many countries around the world due to the huge economic value of the total natural fiber. Verticillium wilt, caused by the soil-borne pathogen *Verticillium dahliae*, is the most devastating disease that led to extensive yield losses and fiber quality reduction in cotton crops. Developing resistant cotton varieties through genetic engineering is an effective, economical, and durable strategy to control Verticillium wilt. However, there are few resistance gene resources in the currently planted cotton varieties, which has brought great challenges and difficulties for breeding through genetic engineering. Further revealing the molecular mechanism between *V. dahliae* and cotton interaction is crucial to discovering genes related to disease resistance. In this review, we elaborated on the pathogenic mechanism of *V. dahliae* and the resistance mechanism of cotton to Verticillium wilt. *V. dahliae* has evolved complex mechanisms to achieve pathogenicity in cotton, mainly including five aspects: (1) germination and growth of microsclerotia; (2) infection and successful colonization; (3) adaptation to the nutrient-deficient environment and competition of nutrients; (4) suppression and manipulation of cotton immune responses; (5) rapid reproduction and secretion of toxins. Cotton has evolved multiple physiological and biochemical responses to cope with *V. dahliae* infection, including modification of tissue structures, accumulation of antifungal substances, homeostasis of reactive oxygen species (ROS), induction of Ca^2+^ signaling, the mitogen-activated protein kinase (MAPK) cascades, hormone signaling, and PAMPs/effectors-triggered immune response (PTI/ETI). This review will provide an important reference for the breeding of new cotton germplasm resistant to Verticillium wilt through genetic engineering.

## 1 Introduction

**Figure 1:**
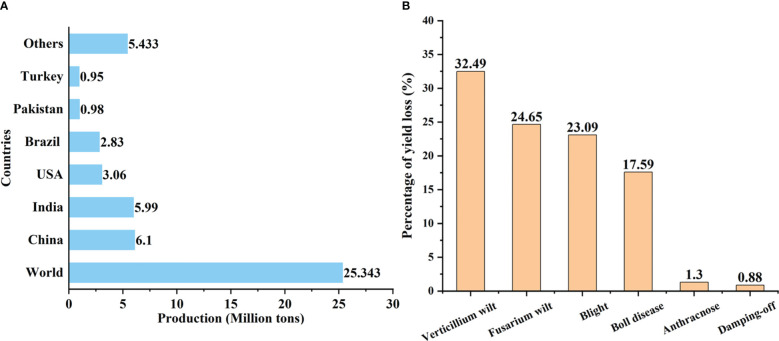
**(A)** Cotton production in major cotton-producing countries in 2022–2023. **(B)** Percentage of yield loss due to the major cotton diseases in China in 2021 (The data were obtained from the Agricultural Technology Extension Service Center of the Ministry of Agriculture and Rural Affairs of China).

Cotton is an extremely important economic crop in the world, as it contributes about 35% of total nature fiber for the textile industry and also serves as one of the sources of edible oil and livestock feed (Man et al., 2022). Cotton is cultivated in more than 80 countries, of which approximately 30 regard cotton as a commercially leading crop (Abdelraheem et al., 2019). Data from the U.S. Department of Agriculture show that the total global cotton production is 25.343 million tons in 2022–2023 (Meyer and Dew, 2023). China was the largest raw cotton producer, followed by India, USA, Brazil, and Pakistan producing 6.1, 5.99, 3.06, 2.83, and 0.98 million tons, respectively (**Figure 1A**). The cotton genera (*Gossypium* spp.) include 45 diploid species (2n = 2x = 26) and seven tetraploid species (2n = 4x = 52). The appearance morphology and fiber characteristics of different cotton genera are quite different, including variable leaf shapes, different fiber characteristics, and diverse plant architectures ranging from wild perennial small trees and shrubs to cultivated annual herbaceous plants (Huang et al., 2021a). Two diploid species *Gossypium herbaceum* (Levant or Arabian cotton) and *Gossypium arboreum* (Desi cotton) and two allotetraploid species *Gossypium barbadense* (Sea Island cotton) and *Gossypium hirsutum* (Upland cotton) are cultivated globally (Egan and Stiller, 2022). Among them, *G. hirsutum* is the most widespread and encompasses 95% of global cotton production and is also the main target of cotton breeding (Baran et al., 2022; Yang et al., 2022).

Being exposed to various environmental cues, a significant decrease in yield and fiber quality is caused by various negative factors such as drought, salinity, temperature stress, pests, nematodes, bacteria, viruses, and fungi (Shaban et al., 2018; Kamburova et al., 2022). Especially, Verticillium wilt, the cancer of cotton crops, is the most devastating disease because of its widespread distribution and strong pathogenicity under favorable conditions. In 2021, the cotton losses in China caused by Verticillium wilt accounted for 32.49% of the total losses caused by different diseases (**Figure 1B**). The average loss recovery rates for five years of Verticillium wilt were much lower than that of other cotton diseases (except cotton boll disease) and pests (Kun and Shuo, 2020). In recent years, Verticillium wilt has become increasingly serious due to climatic variation, long-term monoculture, and frequent introduction of new cotton varieties/hybrids in various countries and regions in the world (Ranga et al., 2020).

In the future, in response to the exploding population of the world and global climate deterioration, the demand for food, fresh water, fiber, and bioenergy of humans will increase significantly (Maryum et al., 2022). Developing resistant cotton varieties through genetic engineering is an effective, economical, and durable strategy to control Verticillium wilt, which is critical to maintaining world agricultural production. The detailed elucidation of the molecular mechanism of *V. dahliae*-cotton interaction will help in discovering genes related to disease resistance. Over the past five years, many reviews have summarized the molecular mechanisms of *V. dahliae*-cotton interaction. Zhang et al. have elaborated on the molecular mechanism of microsclerotia development and systemic infection of *V. dahliae* (Zhang et al., 2022e). The molecular mechanism of cotton resistance to Verticillium wilt has also been revealed (Song et al., 2020; Man et al., 2022). However, the detailed mechanism by which *V. dahliae* successfully colonizes the host plant and causes the symptoms of Verticillium wilt in cotton still needs to be further elucidated. At the same time, the molecular mechanism of cotton resistance to Verticillium wilt also needs a comprehensive and detailed elaboration. Therefore, we comprehensively summarized the pathogenic mechanism of *V. dahliae* and the resistance mechanism of cotton to Verticillium wilt in this review. In particular, we provide additional detail on how *V. dahliae* adapts to nutrient-deficient environments of host plants, manipulates host immunity, and causes Verticillium wilt symptoms. In addition, we elaborated the molecular mechanism of cotton resistance to Verticillium wilt through several aspects to facilitate readers to systematically understand the molecular mechanism of cotton disease resistance.

## 2 Verticillium wilt

**Figure 2:**
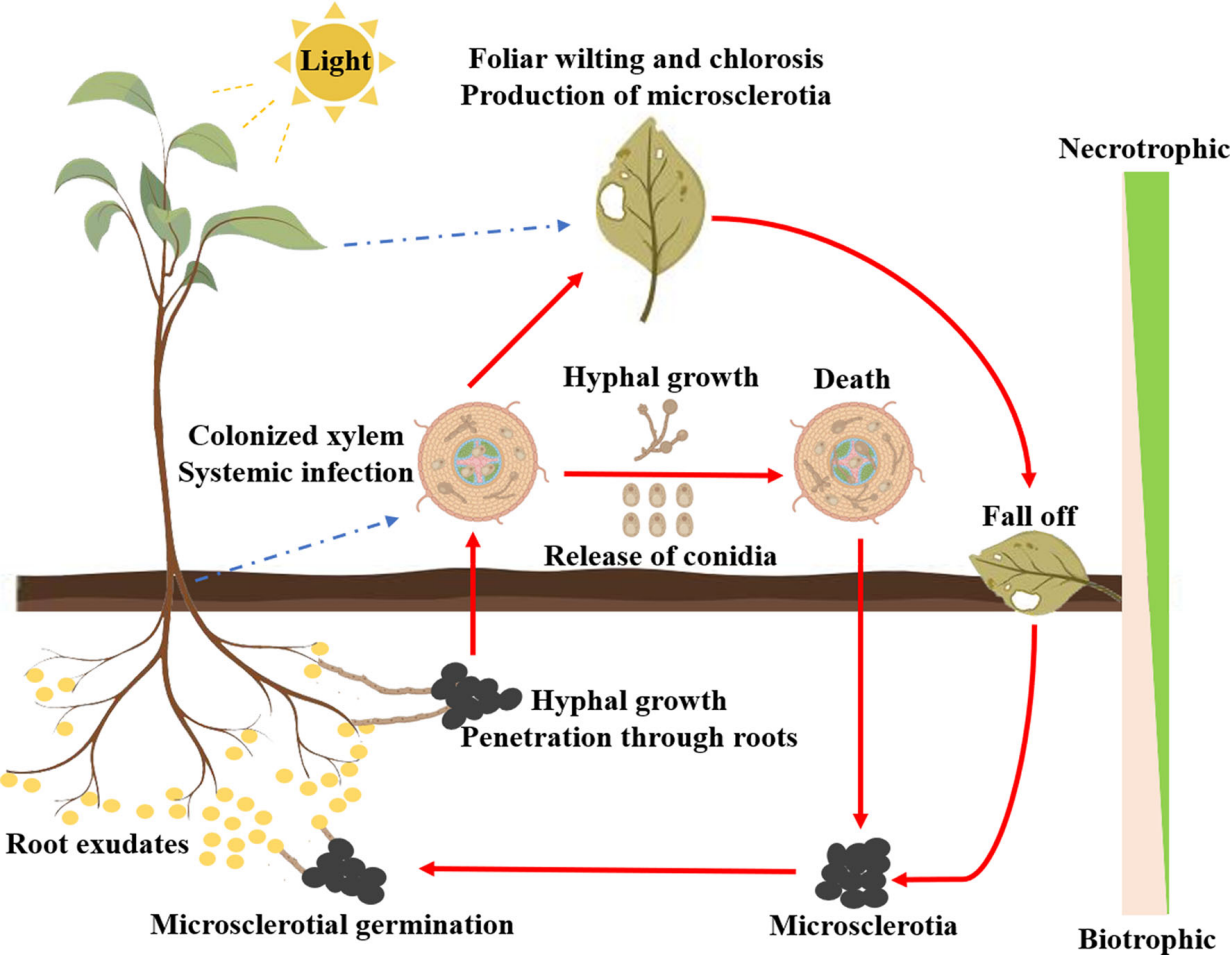
Infection cycle of *V. dahliae* in generic host plants.

Verticillium wilt is among the most devastating plant diseases infecting a broad range of herbaceous annuals and woody perennials, such as cotton, potato, tomato, okra, eggplant, lettuce, spinach, alfalfa, watermelon, strawberry, oilseed rape, sunflower, olive, maple, and smoke-tree (Fradin and Thomma, 2006; Dhar et al., 2020; Wu et al., 2022). *V. dahliae* is the leading cause of Verticillium wilt and its resting body microsclerotia can survive for up to 14 years in the absence of a host or under adverse conditions (Short et al., 2015). Upon sensing the signal from the root exudates of the host plants, the microsclerotia germinate into hyphae and infect through the root tips, lateral roots, or wounds of the host plants (Fradin and Thomma, 2006). After reaching the xylem, the hyphae spread systemically along the vascular system. During the infection of the host plants, *V. dahliae* sequentially undergoes the biotrophic and necrotrophic stages (Lo Presti et al., 2015). In the biotrophic stage, *V. dahliae* draws its nutrients from the host plants for the production of conidia and systemic infection. A large number of hyphae and conidia colonize the xylem resulting in foliar wilting and chlorosis and even death of the host plants (de Sain and Rep, 2015; Zhang et al., 2022b). With the death of the host plants, *V. dahliae* enters the necrotrophic phase and eventually forms microsclerotia to ensure long-term survival (**Figure 2**).

Many environmental factors can affect the incidence of cotton Verticillium wilt, such as the number of microsclerotia in the soil and the diseased plant residues, photoperiod, light intensity, temperature, humidity, irrigation methods, and cultivation techniques (Karademir et al., 2012; Shaban et al., 2018; Wang et al., 2023). The symptoms of cotton infected by *V. dahliae* are yellowing and necrotic leaves, browning of vessels, and wilting. Under the favorable temperature and humidity, the *V. dahliae* in infected cotton will produce white spores to promote the spread of Verticillium wilt, resulting in the rapid death of the cotton (Fradin and Thomma, 2006). *V. dahliae* can also penetrate the bolls and seeds, and the infected seeds are conducive to the further spread of *V. dahliae*. The micronaire and span length of the fiber was seriously reduced in infected cotton (Zhang et al., 2012).

## 3 Pathogenic mechanism of *V. dahliae*

The pathogenic molecular mechanism of *V. dahliae* is relatively sophisticated and controlled by multiple signaling pathways. The main mechanism that causes symptoms of Verticillium wilt is vascular occlusion and the production of toxins. As *V. dahliae* penetrates the xylem vessels, the massive mycelium and plant defense-driven structures and biomacromolecules produced by parenchyma cells block the vessels, interfering with the transportation of water and nutrients in plants (Zhang et al., 2022b). The water imbalance occurs in the host plants and causes wilting and yellowing of leaves and even death (Song et al., 2020). In the toxin hypothesis, the toxins produced by *V. dahliae* have been shown to act on the cell walls, plasma membrane, microfilaments, microtubules, and other intracellular components, leading to cytotoxicity, rapid destruction of cell walls, and disordered host defense responses (Chen et al., 2016a; Zhao et al., 2020; Zhang et al., 2022b). The growth and development and successful colonization of *V. dahliae* are the prerequisites for the occurrence of Verticillium wilt. In brief, both the germination and growth of microsclerotia, attachment and infection to the host roots, degrading the host cell walls, adapting to the nutrient-deficient environment and competing for nutrients, manipulating and evading host immunity, and the secretion of toxins affect the development of Verticillium wilt.

### 3.1 Development and germination of microsclerotia

The key steps of *V. dahliae* infection include germination of microsclerotia, hyphae penetrating the root epidermis, invading hyphae extending in the intercellular space of the root cortex, colonization of hyphae in vessels, production of the conidia to promote the vertical systemic reproduction, and the free passage of the hyphae through the intertracheary pits to cross adjacent xylem vessels to achieve horizontal colonization (Zhao et al., 2014; Tian and Kong, 2022; Zhang et al., 2022b). As the long-term resting structures, germination of microsclerotia is an important step in the occurrence of Verticillium wilt, which is affected by the air, temperature, humidity, soil organic matter content, and pH (Yang et al., 2004; Shaban et al., 2018). The G protein receptors, Ca^2+^, small GTPases, and cAMP were involved in the germination and development of microsclerotia (Luo et al., 2019). VdPbs2 (mitogen-activated protein kinase kinase), VdSkn7 (two-component stress response regulator), VdOCH1 (α-1,6-mannosyltransferase), and VdAda1 (Ada1 subunit) positively regulated the formation of microsclerotia, and the deletion of those genes reduced the pathogenicity of *V. dahliae* (Tian et al., 2016; Tang et al., 2017; Zhang et al., 2019b; Geng et al., 2022). However, some studies suggested that the production of microsclerotia is negatively correlated with the pathogenicity of *V. dahliae*. The deletion mutant strains of *V. dahliae* with *ΔVdPKAC1* (cAMP-dependent protein kinase A), *ΔVGB* (G protein β subunit), *ΔVdMsn2* (C2H2 transcription factor), or *ΔVdPLP* (patatin-like phospholipase) have increased production of microsclerotia but reduced the pathogenicity to host plants (Tzima et al., 2012; Tian et al., 2017; Qi et al., 2018).

### 3.2 Colonization in the roots of host plants

Before penetration of the roots of host plants, the secretome of *V. dahliae* exerts toxicity to suppress the growth of antagonistic bacteria in the rhizosphere environment to ensure *V. dahliae* survival and eventual colonization (Snelders et al., 2020; Snelders et al., 2021). To colonize the host plants, *V. dahliae* needs to successfully adhere to and penetrate the root of the host plants. The hyphae of *V. dahliae* surrounding the roots tightly adhere to the root epidermis and then form the hyphopodium at the infection site, which develops into penetration pegs piercing the root epidermis and cortical cells to further infection (Zhang et al., 2022b; Zhang et al., 2022e).

A variety of genes are involved in the infection process of *V. dahliae* in host plants. The deletion strains with *VdBre1* (encoding a ubiquitin ligase) showed dramatically reduced penetration ability and nonpathogenic symptoms in cotton (Wang et al., 2021c). The nuclear transcription factor Som1 was crucial for adhesion and penetration to the roots, while the nuclear transcription factor Vta3 was required in the colonization of root surfaces (Bui et al., 2019). As a positive regulator of hyphopodium formation, *VdSte11*, a homolog gene of mitogen-activated protein kinase kinase kinase, was critical for penetrating host plants (Yu et al., 2019). The cellophane surface-induced gene *VdCSIN1* regulated the formation of hyphopodium *via* the cAMP-mediated signal pathway to promote the colonization of the host plants (Sun et al., 2019). The sterol C-8 isomerase VdERG2 played a crucial role in the growth and penetration of mycelium on cellophane and knockout of the *VdERG2* gene impaired the pathogenicity of *V. dahliae* (Lv et al., 2022a). While the osmosensor VdSho1 regulated the ability to penetrate the plant through the MAPK pathway (Li et al., 2019b). The velvet protein Vel1 was required for the formation and distribution of conidia in the xylem and for controlling the form of hyphae during the first phases of plant colonization (Höfer et al., 2021).

Penetration pegs are an important tool for *V. dahliae* to penetrate the host roots. The plasma membrane-co-located proteins VdNoxB (catalytic subunit of membrane-bound NADPH oxidase) and VdPls1 (tetraspanin) mediated ROS production, which activated VdCrz1 (calcineurin-responsive zinc finger transcription factor) signaling through Ca^2+^ elevation in hyphopodia to regulate the formation of penetration pegs (Zhao et al., 2016). The penetration pegs further develop into the hyphal neck, which separates the hyphopodium from the invasive hyphae and forms a fungal-host interface to facilitate the delivery and secretion of small secreted proteins (Jin et al., 2021). The cytoskeleton protein VdSep5 was critical to the septin-ring-organized hyphal neck, while the vesicular trafficking factors VdSec22 and VdSyn8 and the exocyst subunit VdExo70 positively regulated the delivery of the secreted proteins to the hyphal neck. The virulence of Vd*Δsep5*, Vd*Δsec22*, Vd*Δsyn8*, and Vd*Δexo70* mutants was significantly reduced to cotton roots (Zhou et al., 2017).

### 3.3 Degradation of the plant cell walls

The plant cell walls, consisting predominantly of cellulose, hemicelluloses (especially xylan), pectin, lignin, and minor structural proteins, are a dynamic structure that plays an important role in preventing the invasion of pathogens (Mielke and Gasperini, 2019; Ishida and Noutoshi, 2022). The cell walls degrading enzymes produced by the pathogens are essential for the colonization of the host plants. Analysis of the genome sequence of *V. dahliae* suggested that there are a large number of cell wall degrading enzymes, including pectinase, xylanase, cellulase, and protease (Chen et al., 2016a). The sucrose nonfermented protein kinase gene *VdSNF1* and the specific secreted protein gene *VdSSP1* positively regulated the activities of cell walls degrading enzymes and were essential for the virulence of *V. dahliae* on host plants (Tzima et al., 2011; Liu et al., 2013). Besides, pathogenesis-related genes *VdPR1* and *VdPR3* affected the pathogenicity of *V. dahliae* by regulating cellulase activity (Zhang et al., 2015; Zhang et al., 2016b). The polygalacturonase VdPG1 and the xylanase VdXyn4 digested pectin and xylan respectively in the cell walls to enhance the pathogenicity of *V. dahliae* to cotton (Liu et al., 2017a; Wang et al., 2021a). Further, the transcription factor VdFTF1 and N-ethylmaleimide-sensitive factor attachment protein receptors VdSec22 and VdSso1 regulated the vesicle trafficking and translocation of pectinases, cellulases, and xylanases (Wang et al., 2018a; Zhang et al., 2018).

### 3.4 Adapting to the nutrient-deficient environment and competing for the nutrients

After penetration to the roots, the invasive hyphae of *V. dahliae* enter the xylem vessels from the intercellular space of the root cortex and rapidly reproduce and invade the vascular bundles. In responding to plant defense responses, *V. dahliae* must adapt to the nutrient-deficient intracellular environment and compete with the host for its nutrients. The glutamate-rich protein VdGARP1 sensed the infertile conditions to promote the transformation of *V. dahliae* from a saprophytic state to microsclerotia for long-term survival (Gao et al., 2010). *VdAsp1*, encoding an inositol polyphosphate kinase, regulated the transition of invasive hyphae from vegetative growth to asexual reproduction to adapt to the nutrient-deficient environment (Tian et al., 2022). The bZIP transcription factor VdAtf1 participated in virulence *via* the regulation of inorganic nitrogen utilization in *V. dahliae* (Tang et al., 2020b). As participants in the acquisition of thiamine, VdThit, VdThi4, and VdThi20 were required for the pathogenicity of *V. dahliae* to host plants (Hoppenau et al., 2014; Qi et al., 2016; Qin et al., 2020). Besides, two M35 family metalloproteinases VdM35-1 and VdASPF2 were involved in the utilization of carbon sources (Lv et al., 2022b). The ferric reductase FreB of *V. dahliae* reduced environmental ferric iron to bioavailable ferrous iron to obtain iron from plant cells and maintained its pathogenicity (Rehman et al., 2018). While VdHapX, a bZIP transcription factor, played a crucial role in iron homeostasis in response to iron-deficient and iron-excess conditions and was involved in the full virulence in *V. dahliae* (Wang et al., 2018b). Under the iron-deficient environment of the xylem, Asp−type small cysteine-rich secretory proteins VdSCPs sequestered ferric iron that further aggravated the deficiency of ferric iron in the xylem, thereby reducing the disease resistance of host plants (Wang et al., 2022a).

### 3.5 Manipulation and suppression of the immune responses of host plants

To successfully colonize and rapidly infect host plants, *V. dahliae* employs complex molecular mechanisms to manipulate and suppress the immune responses of host plants. Two superoxide dismutases VdSOD1 and VdSOD5 were nonessential for the normal vegetative growth of *V. dahliae*, but regulated the detoxification of both extracellular ROS generated from the host and intracellular ROS produced by the normal metabolism of *V. dahliae* (Tian et al., 2021a; Tian et al., 2021b). Besides, Chr2g00380 (cytochrome P450 monooxygenases), VdDpb4 (histone-fold protein of the ISW2 chromatin remodeling complex), and three transcription factors VdAtf1, VdYap1, and VdSkn7 were involved in responding to ROS stress produced by the host plants (Tang et al., 2020a; Wang et al., 2020c; Zhang et al., 2022c). During the infection, the nonribosomal peptide synthetase VdNPS suppressed the expression of PR genes, production of ROS, and SA-mediated signaling of host plants to enhance the pathogenicity of *V. dahliae* (Luo et al., 2020). While the Alt a 1 family protein PevD1 from *V. dahliae* inhibited the antifungal activity of the pathogenesis-related protein GhPR5 to overcome the host defense system (Zhang et al., 2019c). The polysaccharide deacetylase VdPDA1 enhanced the deacetylation of chitin oligosaccharides, leading to impaired ability of host plants to recognize chitin oligosaccharides, thereby inhibiting the host plant immune response (Gao et al., 2019). As a candidate effector, VdCE11 contributed to pathogenicity in cotton and Arabidopsis by enhancing the accumulation and activity of the aspartic proteases, which were negative regulators of immunity from cotton and Arabidopsis (Li et al., 2023).

The small RNAs (sRNAs) can deliver between filamentous pathogens and host plants to trigger transkingdom RNA silencing or RNA interference (RNAi) in recipient cells, thereby altering plant defenses and pathogen virulence (Huang et al., 2019; Zhao et al., 2021). The small RNA VdrsR-1 modulated the floral transition of host plants and prolonged the vegetative growth of host plants thereby favoring the propagation of *V. dahliae* (Zhang et al., 2022a). The secretory silencing repressor VdSSR1 from *V. dahliae* can translocate to the plant nucleus and inhibite the nucleocytoplasmic shuttling of sRNA. VdSSR1 increased the virulence of *V. dahliae* in plants by suppressing the accumulation of mobile plant miRNAs in fungal cells to prevent subsequent transkingdom silencing of virulence genes (Zhu et al., 2022a).

### 3.6 Leading to necrosis, wilting, and defoliation

Although some studies have shown that the crude extracts of *V. dahliae* can cause the collapse of microfilaments and microtubules in plant cells, the physiological and biochemical mechanism leading to wilting and defoliation of the host plants is still unclear. Previous studies have shown that the necrosis- and ethylene-inducing-like protein VdNLP caused foliar necrosis in host plants (Wang et al., 2004). The proposed mechanism was that VdNLP interacted with glycosylinositol phosphorylceramide (GIPC) sphingolipids to form complexes with terminal monomeric hexose moieties of GIPCs that insert into the plant plasma membrane (Lenarčič et al., 2017). Now, VdNEP (VdNLP1) was suggested as a sensitive molecular marker to distinguish the defoliating and nondefoliating *V. dahliae* strains (Triantafyllopoulou et al., 2022). A cytochrome P450 monooxygenase VdCYP1 regulated at least 14 kinds of secondary metabolites syntheses in *V. dahliae* and among them, sulfacetamide could induce necrosis and wilting symptoms in cotton (Zhang et al., 2016a). As a homologous protein of N-acylphosphatidylethanolamine-hydrolyzing phospholipase D, VdDf7 was involved in the generation of N-lauroylethanolamines (NAEs). Excessive synthesis of NAEs in *V. dahliae* induced the overexpression of fatty acid amide hydrolase and disrupted NAEs metabolism in cotton, finally causing defoliation by altering sensitivity to abscisic acid (Zhang et al., 2019a). While the elicitor PevD1 can target the NAC transcription factor ORE1 in Arabidopsis or cotton to manipulate ethylene biosynthesis that triggered *V. dahliae*-induced leaf senescence (Zhang et al., 2021).

## 4 Molecular mechanisms of cotton resistance to Verticillium wilt

**Figure 3:**
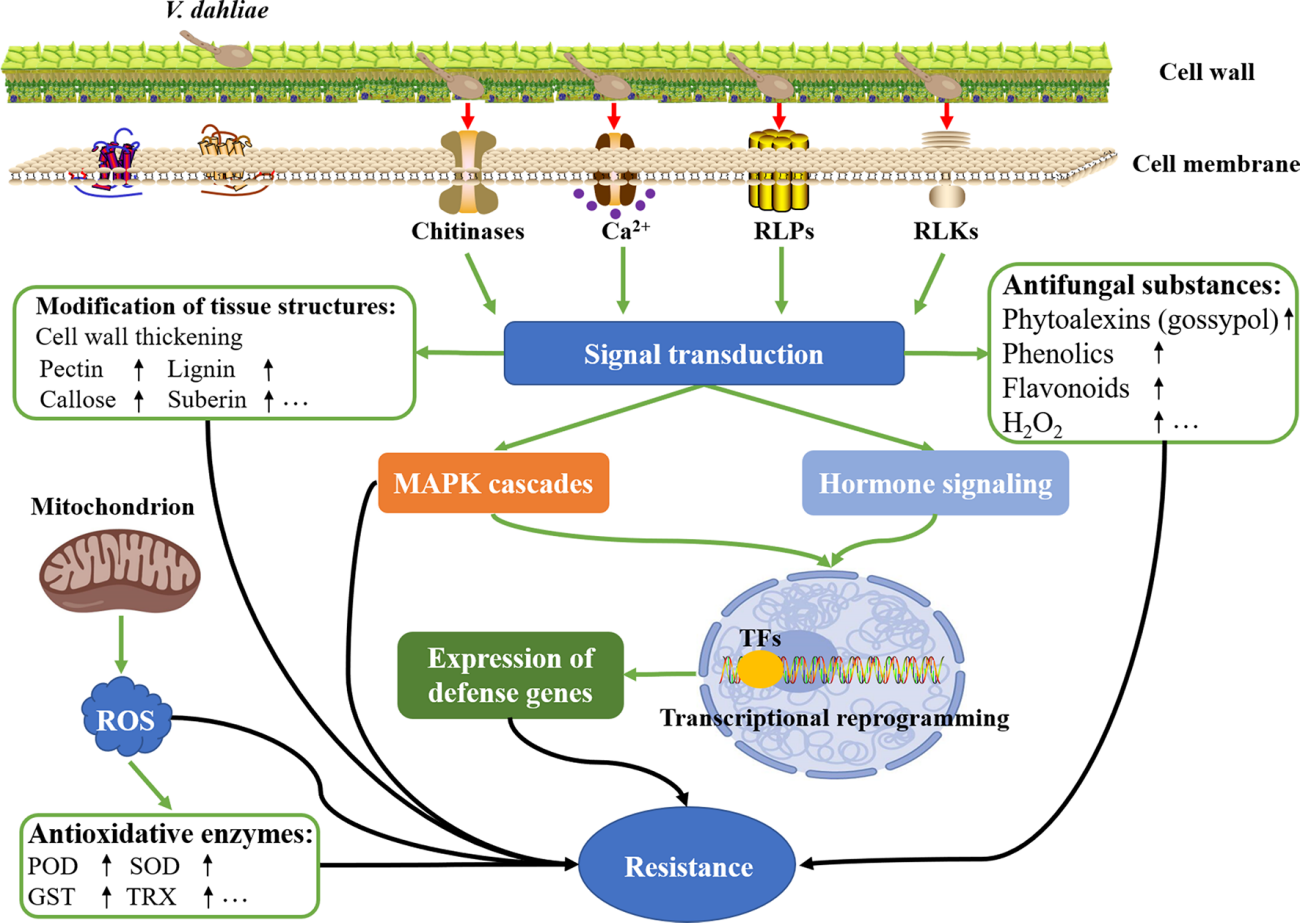
Molecular mechanisms of cotton resistance to *V. dahliae* infection. Upon *V. dahliae* infection, a series of physiological and biochemical responses regulated by the sophisticated molecular mechanism was activated, thereby initiating the resistance to Verticillium wilt in cotton plants.

In response to *V. dahliae* infection, various physiological and biochemical characteristics in cotton will change accordingly to ensure the plants survive under such stressful conditions. Both the physiological and biochemical resistance is mediated by the complex molecular mechanism. In recent years, with the rapid development of molecular biotechnology, breakthroughs have been made in the study of the molecular mechanism of the interaction between *V. dahliae* and cotton. The molecular mechanisms of cotton resistance to Verticillium wilt mainly include the following aspects, such as modification of tissue structures, accumulation of antifungal substances, homeostasis of ROS, secretion of oxidoreductase and hydrolase, production of receptor-like proteins and kinases, regulation of transcription factors, activation of hormone signaling, and induction of hypersensitive response (HR) and the development of systemic acquired resistance (SAR) (**Figure 3**) (Song et al., 2020; Wu et al., 2022). Different defense signals are regulated by different resistance-related genes, and multiple defense signals are often intertwined into a complex signaling network.

### 4.1 Modification of tissue structures

To protect cell walls from being degraded by *V. dahliae*, cotton will modify the cell walls or inhibit the cell wall degrading enzymes from *V. dahliae* by defense-related protein. As the main component of the cell walls, pectin is involved in the plant defense responses against *V. dahliae*. The polygalacturonase VdPG1 could digest the pectin of cell walls, while the polygalacturonase-inhibiting protein GhPGIP1 inhibited the activity of VdPG1 to ensure the integrity of the cell walls and enhanced the resistance of cotton to *V. dahliae* (Liu et al., 2017a; Liu et al., 2017b). Pectin methylesterase GhPME2/GhPME31 catalyzed the demethylation of pectin, resulting in pectin being more easily hydrolyzed by VdPG1. The pectin methylesterase inhibitor GhPMEI3 inhibited the activity of GhPME2/GhPME31 and enhanced the degree of methyl esterification of pectin thereby protecting the cell walls from degradation (Liu et al., 2018). Moreover, the long non-coding RNA lncRNA7 and its regulating gene *GbPMEI13* positively regulated the accumulation of high-methyl esterified pectin in cell walls and enhanced cotton resistance to *V. dahliae* (Zhang et al., 2022d). Chang et al. verified that the galactosyltransferase gene *GhRFS6* positively regulated cotton resistance to Verticillium wilt through involvement in pectin and cell wall synthesis (Chang et al., 2023). Proteomic analyses on xylem sap suggested that the stem mechanical strength and accumulation of cell wall-related proteins significantly enhanced in the resistant cotton varieties after *V. dahliae* infection, but not in the susceptible varieties (Yadav et al., 2020).

During the infection of *V. dahliae*, the deposition of lignin, callose, and suberin occur rapidly to limit or delay the invasion of *V. dahliae*. The lignin and callose deposition increased in resistant and susceptible cotton varieties after *V. dahliae* infection, but the increase was more pronounced in resistant cotton varieties (Xu et al., 2011; Zhang et al., 2017). It has been reported that GhBOP1 (BLADE-ON-PETIOLE1), the laccase GhLAC15, the ribosomal protein L18A GhARPL18A-6, the cinnamyl alcohol dehydrogenases GhCADs, the nonspecific lipid transfer protein GhnsLTPsA10, and transcription factors GbERF1-like, GhWRKY1-like, and GhODO1 enhanced the cotton resistance to *V. dahliae* through functioning as the positive regulators of lignin biosynthesis (Guo et al., 2016; Zhang et al., 2019d; Zhang et al., 2019e; Zhang et al., 2019; Chen et al., 2021a; Hu et al., 2021; Li et al., 2022; Zhu et al., 2022b). Suberin is deposited in the cell walls of root endodermis, outer cortex, periderm, and other marginal tissues, forming another physical barrier to resist the invasion of pathogens (Xin and Herburger, 2021). GbCYP86A1-1, a cytochrome P450 fatty acid ω‐hydroxylase, was involved in the formation of suberin and positively regulated the resistance of cotton and Arabidopsis to *V. dahliae* (Wang et al., 2020a). Overexpression of the pathogenesis-related protein gene *GbPR10.5D1* in cotton enhanced the resistance to *V. dahliae*, accompanied by the activation of genes involved in suberin biosynthesis (Guo et al., 2022).

### 4.2 Accumulation of antifungal substances

The antifungal substances including phytoalexins, phenolics, and flavonoids of cotton accumulate to inhibit the spore germination and germ tube elongation of *V. dahliae*. As the toxic phytoalexins, the deoxyhemigossypol (dHG), hemigossypol (HG), desoxy-6-methyoxyhemigossypol (dMHG), and 6-methoxyhemigossypol (MHG) played a vital role to kill the conidia and mycelia as well as inhibited the sporulation of *V. dahliae* (Wagner et al., 2015). Silencing two major melatonin biosynthesis genes *GhSNAT1* (serotonin N-acetyltransferase) and *GhCOMT* (caffeic acid O-methyltransferase) compromised cotton resistance to *V. dahliae* accompanied by reduced gossypol levels (Li et al., 2019a). Phenolics such as caffeic acid and ferulic acid significantly inhibit the growth of *V. dahliae* colony, while the flavonoids such as naringenin, quercetin, and dihydroquercetin could inhibit the growth of *V. dahliae* mycelia (Hu et al., 2018a; Xiong et al., 2021a). Compared with the control cotton variety S78, the spontaneous mutant cotton with red coloration (S156) has increased resistance to *V. dahliae* due to the increased flavonoid content and gene expressions of flavonoid biosynthesis (Long et al., 2019). Luo et al. found that the biosynthetic pathway of flavonoids was activated under phosphate-deficient conditions, thereby enhancing the resistance of cotton to *V. dahliae* (Luo et al., 2021). Moreover, GhWRKY41 positively regulated the cotton resistance to *V. dahliae* by enhancing the accumulation of flavonoids (Xiao et al., 2023).

### 4.3 PAMP- and effector-triggered immunity

To defend against pathogen invasion, plants can employ the innate immune system to sense specific molecules and initiate subsequent resistance responses. The first tier is based on the cell surface pattern recognition receptors (PRRs) to recognize pathogen/microbe-associated molecular patterns (PAMPs/MAMPs), resulting in PAMPs-triggered immune response (PTI) (Ramirez-Prado et al., 2018). A growing amount of PAMPs/MAMPs has been identified, such as bacterial flagellin, lipopolysaccharides, elongation factor Tu, peptidoglycan, fungal chitin, xylanase, and oligogalacturonides derived from the plants (Zhu et al., 2022c). Successfully invaded pathogens have evolved to generate diverse effector proteins to inhibit PTI. While plants can perceive effectors from pathogens through transmembrane or intracellular receptors (R proteins) to initiate the second tier of defense, which is called effector-triggered immunity (ETI) (Ramirez-Prado et al., 2018). Activation of PTI or ETI triggers numerous overlapping cell signaling events, including Ca^2+^ fluxes, production of ROS, MAPK cascades, transcriptional reprogramming, and hormone biosynthesis (Köster et al., 2022).

As a typical PAMP, chitin released from fungal cell walls by plant chitinases can trigger the plant immune responses (Ramirez-Prado et al., 2018). The infection of *V. dahliae* triggered the secretion of chitinases to degrade the fungal cell wall and the cotton chitinases 23, 28, 32, and 47 have been shown to positively regulate resistance to Verticillium wilt (Xu et al., 2016; Han et al., 2019). To suppress the chitin signaling pathway, the serine protease VdSSEP1 secreted from *V. dahliae* could hydrolyze chitinase 28. While the cysteine-rich repeat protein CRR1 protected chitinase 28 from cleavage by VdSSEP1. Thus, silencing either chitinase 28 or CRR1 in cotton reduced resistance to Verticillium wilt, whereas overexpression of CRR1 in cotton enhanced its resistance (Han et al., 2019).

Receptor-like proteins (RLPs) and receptor-like kinases (RLKs) are important PRRs with distinct extracellular domains for the perception of different ligands (He et al., 2018c). As the leucine-rich repeat receptor-like protein (eLRR-RLP), *Ve1* is responsible for resistance to *Verticillium* spp in tomato, Arabidopsis, tobacco, and cotton (Fradin et al., 2011; Song et al., 2018). The *Ve* homologous genes *GbVe1*, *GbaVd1*, *GbaVd2*, *Gbvdr3*, *Gbvdr5*, and *Gbvdr6* also conferred resistance to Verticillium wilt in Arabidopsis and cotton (Li et al., 2015; Chen et al., 2016b; Chen et al., 2017; Yang et al., 2017). Another large class of receptor-like proteins is nucleotide-binding site-leucine rich repeat (NBS-LRR) proteins that contain a central NBS and a C-terminal LRR domain. According to the diversity of N-terminal structures, NBS-LRR proteins are further divided into CC-NBS-LRR (CNL) and TIR-NBS-LRR (TNL) families. The CNL genes *GbRVd* and *GbCNL130* promote resistance to Verticillium wilt by activating the SA signaling pathway and strong accumulation of ROS (Yang et al., 2016; Li et al., 2021). While another CNL gene *GbaNA1* mediated resistance to *V. dahliae* by activating the production of ROS and activation of the Eth signaling pathway (Abreu et al., 2018). Furthermore, the transfer of TNL gene *GhDSC1* to *dsc1 A. thaliana* mutant conferred resistance to *V. dahliae* and this resistance was coupled with ROS accumulation and activation of the JA signaling pathway (Li et al., 2019d).

As another important PRRs, RLKs are divided into more than 21 subfamilies according to the extracellular ligand binding domain, including leucine-rich repeats (LRRs), lectin, lysin motif (LysM), cysteine-rich receptor-like kinases (CRKs), and wall-associated kinases (WAKs) (Feng et al., 2021). Many pieces of literature have confirmed that receptor-like kinases are involved in the defense response of host plants to *V. dahliae*. The defense-related RLK GbSOBIR1 could phosphorylate GbbHLH171 and play a critical role in cotton resistance to *V. dahliae* (Zhou et al., 2019). While the lysin-motif receptor kinases Lyk1, Lyk2, Lyk7, Lyp1, and LysMe3 played important roles in chitin perception and positively regulate cotton resistance to *V. dahliae* through chitin signaling (Gu et al., 2017; Xu et al., 2017). Two cysteine-rich receptor-like kinases CRK5 and CRK22 played positive regulators in defense responses to *V. dahliae* toxins in Arabidopsis plants by mediating the MPK3/6-WRKY70-TGA2/6-NPR1/3/4-SA signaling pathways (Zhao et al., 2022). In addition, the WAKs also positively regulated cotton response to *V. dahliae* infection (Wang et al., 2020b; Feng et al., 2021; Yang et al., 2021).

### 4.4 Homeostasis of ROS

ROS plays a crucial role in the initial stages of abiotic and biotic stress sensing and contributes to the establishment of subsequent defenses, such as the reinforcement of cell wall structures, hormonal signaling, HR, and SAR (Qi et al., 2017; Mittler et al., 2022). Plant NADPH oxidases (NOXs), also known as respiratory burst oxidase homologs (rbohs), play a predominant role in the metabolic network of ROS. GbRboh5/18 and GhRbohD have been reported to activate ROS production and enhance cotton resistance to Verticillium wilt (Chang et al., 2020; Huang et al., 2021b). However, excessive ROS impairs many cellular functions by altering the structure and function of multiple proteins and causing oxidative damage to DNA, RNA, and membrane lipids (Phua et al., 2021; Mittler et al., 2022). The antioxidative enzymes of plants can scavenge ROS and maintain homeostasis of ROS, including superoxide dismutase (SOD), peroxidase (peroxidase, POD), glutathione S-transferase (glutathione S-transferase, GST), glutathione peroxidase (GPX), and thioredoxin (TRX) (Shaban et al., 2018; Phua et al., 2021). GST, POD, and SOD were involved in the homeostasis of ROS during *V. dahliae* infection so that cotton can prevent itself from being damaged due to excessive accumulation of ROS when initiating defense responses (Li et al., 2019c; Li et al., 2019e; Pei et al., 2019). Moreover, the thioredoxin GbNRX1 regulated the rapid balancing of redox to maintain the homeostasis of apoplastic ROS after infection of *V. dahliae*, which was important for the apoplastic immune response of cotton (Li et al., 2016). Besides, the microRNA miR398b mediated the cleavage of mRNAs of genes that function in ROS homeostasis and caused excessive ROS accumulation, thereby reducing the resistance to *V. dahliae* in miR398b-overexpressing cotton (Miao et al., 2022). As an important antioxidant, anthocyanins also have an effect to maintain the homeostasis of ROS. The silencing of the anthocyanin synthase gene *GbANS* reduced the anthocyanin content in cotton, resulting in excessive accumulation of H_2_O_2_ and attenuating cotton resistance to *V. dahliae* (Long et al., 2018).

### 4.5 Ca^2+^ signaling

The Ca^2+^ signaling is an important messenger of the plant immune system and builds an extremely complicated network of many interconnected nodes, transferring external or internal danger signals to activate multiple defense responses (Jiang and Ding, 2023). The MYB transcription factor GhMYB108 positively regulated cotton resistance to *V. dahliae* by interacting with the major Ca^2+^ sensor GhCML11 (calmodulin-like protein). Further analysis found that Ca^2+^ was beneficial for GhCML11 to enhance the transcriptional activity of GhMYB108, indicating that Ca^2+^ played an important role in the defense response against *V. dahliae* mediated by GhMYB108-GhCML11 (Cheng et al., 2016). Recently, Sun et al. demonstrated that PAMPs activated the expression of the calcium sensor TOUCH 3 (TCH3, also named CML12), which interfered with the auto-inhibitory region of calcium-dependent protein kinases5 (CPK5) and promoted CPK5-mediated phosphorylation of CAM-BINDING PROTEIN 60-LIKE G (CBP60g). The phosphorylation of CBP60g enhanced its transcription factor activity and positively regulated the defense against *V. dahliae* (Sun et al., 2022). In addition, the accumulation of Ca^2+^ induced by *V. dahliae* promoted the acetylation of the calmodulin GhCaM7, thereby activating JA and ROS defense signaling pathways and changing cell osmotic potential to enhance cotton resistance to Verticillium wilt (Zhang et al., 2023a).

### 4.6 MAPK cascades

MAPK cascades are highly conserved in eukaryotes and widely used to amplify and translate environmental and developmental signals into complex physiological and biochemical reactions. The rapid activation of MAPK cascades typically relays and amplifies PTI/ETI-induced downstream signals, such as transcriptional recombination and hormone signaling (Zhang and Zhang, 2022). The typical MAPK cascades are composed of MAPKs (MPKs), MAPK Kinases (MAPKKs/MKKs/MEKs), and MAPK Kinase Kinases (MAPKKKs/MEKKs). Previous studies have shown that silencing of *GhMKK2*, *GhMKK4*, *GhMKK6*, *GhMKK9*, *GhMPK9*, *GhMPK13*, and *GhMPK25* compromised cotton resistance to the infection by *V. dahliae*, whereas silencing of *GhMKK10* increased resistance to *V. dahliae* (Gao et al., 2011; Zhang et al., 2014; Meng et al., 2018). Moreover, the phosphorylation of the MPK homolog GhNTF6 activated ROS production, callose deposition, and the JA signaling pathway to increase the resistance to *V. dahliae* infection (Zhou et al., 2022). While the overexpression of *GbMPK3* in cotton activated the SA signaling transduction but reduced resistance to *V. dahliae* (Long et al., 2020).

### 4.7 Hormone signaling

Upon pathogen attack, phytohormone signaling is the primary perception of plants and then the subsequent defense signaling network was activated or repressed, including the elicitation of PR genes, the reinforcement of cell wall, production of phytoalexins, induction of SAR, ROS, and MAPK cascades (Dhar et al., 2020; Billah et al., 2021). Salicylic acid (SA), jasmonic acid (JA), ethylene (Eth), auxin (AUX), cytokinin (CTK), gibberellic acid (GA), abscisic acid (ABA), brassinosteroids (BR), and strigolactones (SLs) have been documented in plant defense responses (Shaban et al., 2018; Dhar et al., 2020). These plant hormones form complex signaling networks that integrate environmental cues and respond to diverse pathogens. There is a complex synergy and antagonism between different hormone signals in response to the invasion of diverse pathogens. While SA and JA are the main players against *V. dahliae*, and their roles are well-established (**Table 1**).

**Table 1:** Genes implicated in the SA, JA, and Eth defense signaling in response to *V. dahliae* infection.

Hormones	Gene name	Regulatory mechanism	Cotton species	Resistance/Susceptibility	References
**SA↑**	*GbEDS1*	Enhanced SA accumulation	*G. barbadense*	Enhanced resistance to *V. dahliae* strain Vd991	(Yan et al., 2016)
**SA↑**	*GhTCP4-like*	Promoted SA biosynthesis by activating *GhICS1* expression	*G. hirsutum*	Enhanced resistance to *V. dahliae* strain Vd991	(Jia et al., 2022)
**SA↑**	*GbTSA1*	Activated SA synthesis	*G. barbadense*	Enhanced resistance to *V. dahliae* strain Vd991	(Miao et al., 2019)
**SA↑**	*GhSAP6*	Activated SAR and enlarged SA signaling	*G. hirsutum*	Enhanced resistance to *V. dahliae* strain Vd991	(Hu et al., 2023)
**SA↑**	*GaGSTF9*	Regulated SA accumulation	*G. arboreum*	Enhanced resistance to *V. dahliae* strain Vd07038	(Gong et al., 2018)
**SA↑**	*GhSAMDC*	Regulated SA signaling	*G. hirsutum*	Enhanced resistance to *V. dahliae* strains T5	(Mo et al., 2016)
**SA↑**	*GhSDH1-1*	Involved in SA signaling	*G. hirsutum*	Enhanced resistance to *V. dahliae* strain V080	(Zhang et al., 2020)
**SA↑**	*GhSSI2s*	Induced PAL-mediated SA signaling	*G. hirsutum*	Enhanced resistance to *V. dahliae* strain Linxi 2-1	(Mo et al., 2021)
**SA↑/JA↓**	*GhVLN4*	Enhanced the SA signaling but suppressed JA signaling	*G. hirsutum*	Enhanced resistance to *V. dahliae* strain Vd991	(Ge et al., 2021)
**SA↑/JA↓**	*GhWRKY70*	Activated the SA signaling but suppressed JA signaling	*G. hirsutum*	Increased susceptibility to *V. dahliae* strain Vd991	(Xiong et al., 2019)
**SA↑/JA↓**	*GhGDH2*	Activated the SA signaling but suppressed JA signaling	*G. hirsutum*	Increased susceptibility to *V. dahliae* strain Vd991	(Xiong et al., 2021b)
**SA↑/JA↑**	*GhGPA*	Activated the SA and JA signaling	*G. hirsutum*	Enhanced resistance to *V. dahliae* strain Linxi 2-1	(Chen et al., 2021b)
**SA↑/JA↑**	*GhRPS6*	Activated the SA and JA signaling	*G. hirsutum*	Enhanced resistance to *V. dahliae* strain Vd080	(Zhu et al., 2021a)
**SA↑/JA↑**	*GhPLDδ*	Activated the SA and JA signaling	*G. hirsutum*	Enhanced resistance to *V. dahliae* strain Vd991	(Zhu et al., 2022c)
**JA↓**	*GhCYP82D*	Suppressed the LOXs-mediated biosynthesis of JA	*G. hirsutum*	Increased susceptibility to *V. dahliae* strain Vd991	(Sun et al., 2014)
**JA↑**	*GhLOX2*	Activated the JA defense signaling	*G. hirsutum*	Enhanced resistance to *V. dahliae*	(Shaban et al., 2021)
**JA↑**	*GhlncLOX3*	Enhanced the JA defense signaling	*G. hirsutum*	Enhanced resistance to *V. dahliae* strain Linxi 2-1	(Wang et al., 2021b)
**JA↑**	*GhPLP2*	Positively regulated the JA biosynthesis	*G. hirsutum*	Enhanced resistance to *V. dahliae* strain Vd991	(Zhu et al., 2021b)
**JA↓**	*GhCPK33*	Suppressed the biosynthesis of JA	*G. hirsutum*	Increased susceptibility to *V. dahliae* strain Vd991	(Hu et al., 2018b)
**JA↓**	*GhJAZ2*	Suppressed the JA defense signaling	*G. hirsutum*	Increased susceptibility to *V. dahliae*	(He et al., 2018b)
**JA↑**	*GhbHLH171*	Activated the JA defense signaling	*G. hirsutum*	Enhanced resistance to *V. dahliae*	(He et al., 2018b)
**JA↓**	*HDTF1*	Suppressed the JA signaling	*G. hirsutum*	Increased susceptibility to *V. dahliae* strain Vd991	(Gao et al., 2016)
**JA↓**	*GhHB12*	Suppressed the JA defense signaling	*G. hirsutum*	Increased susceptibility to *V. dahliae*	(He et al., 2018a)
**JA↓**	*GhBLH7-D06*	Suppressed the JA defense signaling	*G. hirsutum*	Increased susceptibility to *V. dahliae* strain Vd991-GFP	(Ma et al., 2020)
**JA↓**	*GhBIN2*	Reduced JA content and suppressed the JA signaling	*G. hirsutum*	Increased susceptibility to *V. dahliae* strain Vd07038	(Song et al., 2021)
**JA↑**	*GhWRKY70*	Activated the JA signaling by interacting with GhAOS	*G. hirsutum*	Enhanced resistance to *V. dahliae* strain linxi2-1	(Zhang et al., 2023b)
**JA↓/Eth↓**	*GhWRKY70D13*	Reduced the content of JA, JA-Ile and ACC	*G. hirsutum*	Increased susceptibility to *V. dahliae* strain Vd991	(Xiong et al., 2020)
**Eth↑**	*GhMPL28*	Activated the Eth defense pathway	*G. hirsutum*	Enhanced resistance to *V. dahliae* strain Vd991	(Yang et al., 2015)

Upward arrows represent the positive regulation of hormone signaling by genes, while downward arrows represent the negative regulation of hormone signaling by genes.

#### 4.7.1 SA signaling in resistance to Verticillium wilt

SA, one of the major defense-related hormones, plays an important role in the activation of the expression of PR genes and establishment of the long-lasting and broad-spectrum disease resistance SAR (Shaban et al., 2018). Although SA has been implicated in defense responses against a wide range of biotrophic and hemibiotrophic pathogens, it plays an auxiliary role against necrotrophic pathogens (Dhar et al., 2020). As a hemibiotrophic pathogen, *V. dahliae* behaves as a biotrophic pathogen during the early stages of infection but it switches to a necrotrophic lifestyle during the later infectious stages. Thus, the SA signaling pathway was required to confer resistance against *V. dahliae*.

The *enhanced disease susceptibility 1* (*EDS1*) gene is a core genetic component in SA-mediated defense responses and is critical for resistance against biotrophic and hemibiotrophic pathogens (Chen et al., 2021c). Silencing of *GbEDS1* in cotton significantly decreased the accumulation of SA and enhanced the susceptibility of cotton to *V. dahliae* (Yan et al., 2016). In plants, the enzymatic reactions for the synthesis of salicylic acid are mainly mediated by phenylalanine lyase (PAL) and isochorismate synthase (ICS). The teosinte branched1/Cincinnata/proliferating cell factor (TCP) transcription factor GhTCP4-like interacted with GhNPR1 to promote GhICS1 expression, leading to accumulation of SA, which was sensed by NPR1 to increase cotton resistance against *V. dahliae* (Jia et al., 2022). As a key player in the elicitation of SA biosynthesis, indole enhances the expression of genes involved in SA defense signaling pathways. Knock-down of *GbTSA1* (tryptophan synthase α) and *GbTSB1* (tryptophan synthase β) enhanced the accumulation of indole, thereby activating SA biosynthesis and defense signaling pathways and improving cotton resistance to *V. dahliae* (Miao et al., 2019). Moreover, the miR530-*GhSAP6* module in cotton leaves responded remotely to *V. dahliae* infection from roots *via* SAR, and then enlarged SA signaling at locations farther from the injection sites, leading to enhanced resistance of cotton plants to *V. dahliae* (Hu et al., 2023).

Moreover, the complex metabolic pathways of plant secondary products often intersect with SA signaling. Gong et al. confirmed that glutathione S-transferase GaGSTF9 positively regulated cotton resistance to *V. dahliae* by maintaining the low-level accumulation of ROS and then affecting SA content (Gong et al., 2018). The S-adenosylmethionine decarboxylase gene *GhSAMDC* encoded key rate-limiting enzymes of spermine biosynthesis, and the constitutive overexpression of *GhSAMDC* in Arabidopsis improved resistance against *V. dahliae* through activating SA signaling (Mo et al., 2016). Additionally, the silencing of succinate dehydrogenase gene *GhSDH1-1* in cotton led to decreased resistance to *V. dahliae* because of the severe damage to the SA-signaling pathway (Zhang et al., 2020). The soluble fatty acid desaturase stearoyl-ACP desaturase regulates the desaturation of fatty acids and produces the monounsaturated fatty acid oleic acid (18:1) in the plastids. Suppressing the expression of *GhSSI2s* (encoding stearoyl-ACP desaturases) reduced the 18:1 level in cotton and autoactivated PAL-mediated SA defense response, thereby enhancing cotton resistance to *V. dahliae* (Mo et al., 2021).

Studies have reported the antagonism interaction between SA and JA/Eth signaling pathways. Overexpression of the actin cytoskeleton gene *GhVLN4* in Arabidopsis enhanced resistance to *V. dahliae* by enhancing the SA defense signaling but suppressing JA defense signaling (Ge et al., 2021). The silence of *GhWRKY70* or *GhGDH2* (encoding glutamate dehydrogenase) led to the suppression of the SA signaling pathway and initiation of the JA signaling pathway, which consequently enhanced the cotton resistance against *V. dahliae* (Xiong et al., 2019; Xiong et al., 2021b). Interestingly, SA and JA signaling pathways are not always antagonistic to each other but also have mutual synergy. The G-protein α-subunit GhGPA positively regulated the resistance of cotton and Arabidopsis plants to *V. dahliae* by activating both SA and JA signaling pathways (Chen et al., 2021b). Knockdown of the ribosomal protein gene *GhRPS6* resulted in decreased SA and JA content and suppressed a series of defensive responses in cotton (Zhu et al., 2021a). The phospholipase GhPLDδ played a positive role in the tolerance to Verticillium wilt through the activation of JA and SA signaling pathways (Zhu et al., 2022c).

#### 4.7.2 JA signaling in resistance to Verticillium wilt

Jasmonates (JAs) is the collective name for JA and its derivatives and belongs to the family of oxylipin compounds, which were produced through oxidation and further conversions of polyunsaturated fatty acids by lipoxygenases (LOXs) and α-LOXs (Cook et al., 2021). JAs play crucial roles in plant responses to biotic and abiotic stresses, especially in defense responses against herbivores, insect pests, wounding, and pathogens (Wasternack and Strnad, 2016; Ghorbel et al., 2021). The JA signaling pathway has been extensively studied in the interaction between *V. dahliae* and cotton. The cytochrome P450 CYP82D (SSN) competed for fatty acids with LOXs and suppressed the LOXs-mediated JA biosynthetic pathway in cotton, thereby weakening the resistance to *V. dahliae* (Sun et al., 2014). Knockdown of *GhLOX2* and *GhlncLOX3* suppressed the expression of JA-related genes and increased cotton susceptibility to *V. dahliae* (Shaban et al., 2021; Wang et al., 2021b). *GhPLP2*, encoding the patatin-like protein, regulated the fatty acid metabolism pools for JA biosynthesis and activated the JA signaling pathway, thereby enhancing the resistance of cotton and Arabidopsis plants to *V. dahliae* (Zhu et al., 2021b). The calcium-dependent protein kinase GhCPK33 phosphorylated the 12-oxophytodienoate reductase GhOPR3, leading to destabilization of GhOPR3, which limited the biosynthesis of JA and decreased cotton resistance to *V. dahliae* (Hu et al., 2018b). Jasmonate-ZIM-domain protein GhJAZ2 inhibited the activity of GhbHLH171, a positive regulator of the JA signaling pathway, resulting in attenuated resistance of *GhJAZ2*-overexpressed cotton to *V. dahliae* (He et al., 2018b).

Many studies have explored the roles of transcription factors involved in the defense system of cotton by modulating JA signaling. As a homeodomain transcription factor, *HDTF1* negatively regulated cotton resistance to *V. dahliae* by inactivating the JA-mediated signaling and JA accumulation, but not affecting SA signaling (Gao et al., 2016). Overexpression of the homeodomain-leucine zipper (HD-ZIP) transcription factor *GhHB12* increased the susceptibility of the cotton to *V. dahliae*, which was associated with the repression of genes expression in JA defense signaling (He et al., 2018a). Silencing of the BEL1-like transcription factor *GhBLH7-D06* enhanced the tolerance of cotton to Verticillium wilt, which was mainly attributed to the activation of lignin biosynthesis and JA defense signaling pathways (Ma et al., 2020). Moreover, the knock-down of the GSK3-like kinase gene *BIN2* (*brassinosteroid insensitive 2*) significantly enhanced the resistance of Arabidopsis and cotton to *V. dahliae* by regulating the endogenous content of JA and the expression of JA-responsive marker genes (Song et al., 2021). Recently, Zhang et al. identified a new WRKY70 (highly homologous to GhWRKY70D02 sequence), which activated the JA defense signaling pathway by interacting with GhAOS, a key enzyme in the biosynthesis of JA, to enhance the tolerance of cotton to Verticillium wilt (Zhang et al., 2023b).

#### 4.7.3 Other hormone signaling in resistance to Verticillium wilt

The ET and JA signaling pathways usually cooperate to defend against *V. dahliae* infection. Silencing of *GhWRKY70D13* led to the increased accumulation of JA, JA-Ile, and ET synthesis precursor ACC and enhanced resistance of cotton to *V. dahliae* (Xiong et al., 2020). By enhancing the transcriptional activity of the ethylene response factor GhERF6, the defense-related major latex protein GhMPL28 activated the ET defense pathway to enhance cotton resistance to *V. dahliae* (Yang et al., 2015). However, there are studies indicating that ET seems to promote the development of Verticillium wilt symptoms. Pantelides et al. confirmed that the perception of ethylene *via* ethylene receptor ETR1 was required in Arabidopsis infection by *V. dahliae* (Pantelides et al., 2010). Overexpression of *AtCTR1* (a negative regulator of ethylene signaling) in cotton led to reduced sensitivity to ethylene but increased resistance to *V. dahliae* (Wang et al., 2022b). The elicitor PevD1 manipulated ethylene biosynthesis in Arabidopsis and cotton and triggered *V. dahliae*-induced leaf senescence (Zhang et al., 2021). Collectively, these results indicated that ET plays a dual role in resistance as well as the development of wilt symptoms.

In the hormone defense signaling network of plants against *V. dahliae*, there are often complex relationships between SA/JA and other hormones. During the infection of *V. dahliae*, the Aux/IAA protein GhIAA43 was a negative regulator of cotton immune response and played a major role in the connection between SA defense signaling and auxin signaling in cotton plants (Su et al., 2022). The APETALA2/ETHYLENE RESPONSIVE FACTOR gene *GhTINY2* positively regulated the resistance of cotton and Arabidopsis plants to *V. dahliae*. Further studies determined that *GhTINY2* fine-tuned the trade-off between immunity and growth by indirectly linking the WRKY51-mediated SA signaling and BZR1-IAA19-regulated BR signaling (Xiao et al., 2021). The carotenoid cleavage dioxygenases GbCCD7 and GbCCD8b positively regulated the accumulation of strigolactone and consequently activated the JA and ABA signaling. The positive feedback loop of ABA and the negative feedback loop of JA could regulate the homeostasis of strigolactone and maintain the balance among these three hormones, thereby improving the tolerance of cotton to Verticillium wilt (Yi et al., 2023).

## 5 Conclusions and perspectives

Since there is a conserved co-evolutionary relationship between plants and pathogens in nature, revealing the pathogenic mechanism of pathogens and the resistance mechanism of host plants will help to improve plant disease resistance. Elucidating the molecular mechanisms of *V. dahliae*-cotton interaction is a key step for the durable and efficient control of cotton Verticillium wilt. However, only a few key molecular mechanisms have been well elucidated. With the release of the genome sequence of *V. dahliae* and cotton, scientific researchers have focused on the molecular mechanisms of *V. dahliae*-cotton interaction, giving us a deep understanding of the complex mechanisms. In this review, we provide a broader picture of the new insights into the interaction between *V. dahliae* and cotton. *V. dahliae* have evolved a variety of approaches to infect cotton, while cotton has developed various defense mechanisms to cope with the threat of Verticillium wilt.

In *V. dahliae*, the molecular mechanism of pathogenicity that has been revealed so far can be roughly divided into five aspects: (1) germination and growth of microsclerotia; (2) infection and successful colonization; (3) adaptation to the nutrient-deficient environment of cotton and competing with cotton for its nutrients; (4) suppression and manipulation of cotton immune response; (5) leading to wilting and defoliation of cotton through rapid reproduction of *V. dahliae* and secretion of toxins. To deal with *V. dahliae*, cotton has evolved a complex defense system, mainly including modification of tissue structures, accumulation of antifungal substances, homeostasis of ROS, induction of Ca^2+^ signaling, the MAPK cascades, hormone signaling, and PTI/ETI. As *V. dahliae* is a hemibiotrophic pathogen generally it is perceived that the defense mechanism of cotton against *V. dahliae* is relatively complex. There are differences in the defense signals dominated by different resistance-related genes and multiple defense signals intersect into a complex signal network.

The pathogenic mechanism of *V. dahliae* and the resistance mechanism of cotton were comprehensively analyzed in this review, providing the target gene resources for effective control of cotton Verticillium wilt. With the development of multi-omics integrative analyses and molecular biology technology, it is possible to mine more key genes in the interaction between cotton and *V. dahliae*, which will be contributed to obtaining cotton varieties that are resistant to Verticillium wilt through genetic engineering and breeding technology.

## Author contributions

YuZ: project administration, writing – original draft, writing – review and editing. MZ: visualization. TL: investigation. LW: visualization. CL: supervision. DL: writing – review and editing. HZ: supervision. YaZ: writing – review and editing. LL: supervision. XG: investigation, supervision. BL: funding acquisition, writing – review and editing. All authors contributed to the article and approved the submitted version.
